# Ectopic tooth in maxillary sinus compressing the nasolacrimal canal: A case report

**DOI:** 10.1097/MD.0000000000025514

**Published:** 2021-05-07

**Authors:** Peng Yang, Hao Liang, Bo Zou, Jianlin Liu, Daoying Yuan, Zhen Meng, Kai Xu

**Affiliations:** a Department of Stomatology; b Precision Biomedical Lab, Liaocheng People's Hospital, Liaocheng University, Liaocheng, China.

**Keywords:** case report, dentigerous cyst, ectopic tooth, endoscope, nasolacrimal duct obstruction

## Abstract

**Rationale:**

Ectopic tooth is tooth erupting out of normal anatomical position. Ectopic tooth can occur in different positions, such as maxillary sinus and nasal cavity. In this article, we present a rare case of an ectopic tooth with a dentigerous cyst in the maxillary sinus compressing the nasolacrimal canal.

**Patient concerns:**

An 8-year-old girl presented with a 2-month history of spontaneous lacrimation in her right eye. When she wept, more tear shed from her right eye than that from the left one. Computed tomographic (CT) imaging showed a huge low-density image containing a tooth in the maxillary sinus in her right maxilla; the right nasolacrimal canal vanished due to the compression of the ectopic tooth.

**Diagnoses:**

Ectopic tooth with dentigerous cyst of right maxilla, and obstruction of nasolacrimal duct

**Interventions:**

The patient underwent nasal endoscopic maxillary sinus cystectomy.

**Outcomes:**

The patient recovered well after cystectomy and has been symptom-free.

**Lessons:**

The unique finding is that this is the first report about ectopic tooth compressing the nasolacrimal canal and inducing spontaneous lacrimation. Treatment: aspect: surgery under endoscope is a minimally invasive approach to ectopic tooth.

## 1 Introduction

Ectopic tooth is tooth erupting out of normal anatomical position. The interaction between the oral epithelium and tooth germ cells plays important roles in tooth development.^[1]^ Abnormal interaction or tooth germ cells migrating to abnormal position caused by developmental disturbance, pathological process or iatrogenic activity during embryonic development may be the pathogenesis of ectopic tooth development.^[2,3]^

Ectopic tooth can occur in different positions, such as maxillary sinus^[3]^ and nasal cavity.^[4]^ Ectopic tooth often induces no symptom originally; however, as tooth development or secondary infection or cyst, symptoms may emerge. The symptoms usually vary according to the anatomical positions of ectopic teeth.

In this article, we present a case of an ectopic tooth with a dentigerous cyst in the maxillary sinus compressing the nasolacrimal canal.

## 2 Case report

This publication of this case was approved by the Ethics Committee of Liaocheng People's Hospital and informed consent was obtained from her parents for the purpose of publication of case details and images.

**Figure 1:**
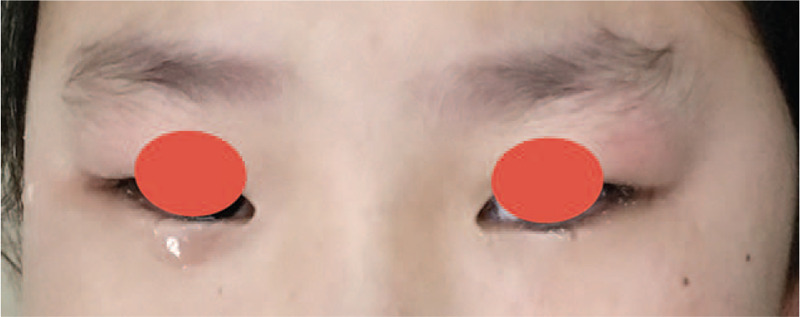
When the patient wept, more tear shed from her right eye than that from the left one.

**Figure 2:**
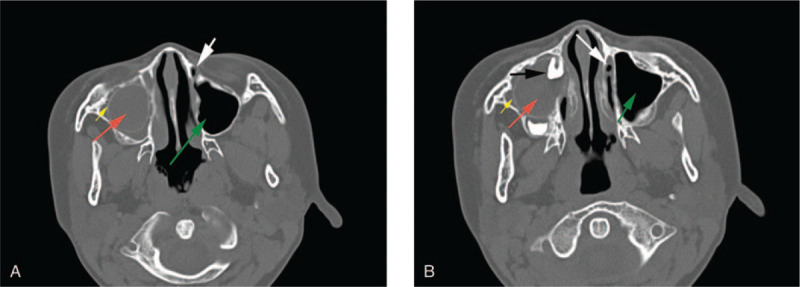
CT imaging of the ectopic tooth, the dentigerous cyst and the blocked nasolacrimal canal. Red arrow indicates the low-density image wrapped by a high density loop (yellow arrow) in the right maxillary sinus; green arrow indicates the left normal maxillary sinus. White arrow indicates the left normal nasolacrimal canal, while at the corresponding right position, the nasolacrimal canal is disappeared. Black arrow indicates a tooth in the cyst.

An 8-year-old girl presented with a 2-month history of spontaneous lacrimation in her right eye. When she wept, more tear shed from her right eye than that from the left one (Fig. 1). Ophthalmologic examination showed that there were no abnormalities in eye bulb and lacrimal gland. Computed tomographic (CT) imaging showed a huge low-density image occupied the location of the maxillary sinus in her right maxilla; the low-density image was wrapped by a high density loop, with an ectopic tooth in (Fig. 2). The superior margin of the lesion was just adjacent to the infraorbital margin, and the inferior margin reached the alveolar process with maxillary deciduous canine and first deciduous molar implicated. Furthermore, compared with the left side, the nasolacrimal canal of the right side vanished due to the compression of the ectopic tooth (Fig. 2). The patient was diagnosed as ectopic tooth with dentigerous cyst of right maxilla, and obstruction of nasolacrimal duct. After receiving a dentigerous cyst cystectomy under endoscope, the patient has been symptom-free for about 3 months. The patient was satisfied with the therapeutic effect

## 3 Discussion

### 3.1 Cause of ectopic tooth

Various factors can induce ectopic tooth, including abnormal development such as cleft palate, benign or malignant lesions, and trauma. Agrawal et al reported a case of intranasal ectopic tooth 1 year after maxillofacial trauma, which was caused by injury-induced tooth bud displacement into the nasal floor.^[5]^ Bhavna reported a mature orbital teratoma in which an ectopic tooth was found^[6]^; besides, Basavaraj shared a case of keratocystic odontogenic tumor containing an ectopic tooth in maxilla.^[7]^ However, as most ectopic teeth, in our case, the cause of the tooth impacted in maxillary sinus was unknown.

### 3.2 Location of ectopic tooth

Most reported locations of ectopic teeth are in maxillary sinus^[3,8–12]^ and intranasal.^[5,13–15]^ Balaji and Reuser respectively reported teeth impacted in the orbit and shared surgical management.^[16,17]^ There are rare cases about a mandibular third molar impacted in the subcondylar region.^[18,19]^ There is another interesting case about an intracranial supernumerary tooth.^[20]^

### 3.3 Symptoms caused by ectopic tooth

Most ectopic teeth have no symptom originally; nevertheless, when they induce secondary infection or compressed important anatomical structures, various symptoms may occur. Ectopic teeth in maxillary sinus or nasal cavity may induce headache and nasal obstruction,^[10,14,20]^ as well as fever and exhaust when there is secondary infection.^[21]^ In our case, the tooth impacted in maxillary sinus, and the symptoms of headache and nasal obstruction were not obvious; however, involuntary tear from the right eye was complained due to the blockage of nasolacrimal duct. To the best of our knowledge, this is the first report about ectopic tooth compressing the nasolacrimal duct.

### 3.4 Diagnosis and treatment of ectopic tooth

Most ectopic teeth were found only when there were symptoms or by accident. Radiological examination is the available method for ectopic teeth diagnose due to their radiopaque image.^[12]^ Panoramic radiograph, Water graphy, cone-beam computed tomography (CBCT), and CT can be used in diagnosing ectopic tooth, while CBCT and CT possess the advantage of clearly revealing the location relationship between the tooth and peripheral anatomies. In our case, CT scan clearly shows the tooth located at the medial wall of sinus and compassed the nasolacrimal duct.

If no symptom, no treatment but clinical follow-up is needed. For symptomatic cases, surgery is the standard and necessary treatment for ectopic teeth. When the tooth was impacted in nasal cavity or maxillary sinus, endoscope can be an available approach for removing the tooth.

## 4 Conclusion

This article shared a rare case of an ectopic tooth with a dentigerous cyst located in the maxillary sinus induced nasolacrimal duct obstruction in a young female manifesting as spontaneous lacrimation from the right eye. CT scan confirmed the diagnosis and location of the ectopic tooth.

Surgery under endoscope is a minimally invasive approach to ectopic tooth.

## Author contributions

**Conceptualization:** Peng Yang.

**Data curation:** Peng Yang, Hao Liang.

**Resources:** Daoying Yuan, Kai Xu.

**Supervision:** Zhen Meng, Kai Xu.

**Writing – original draft:** Bo Zou, Jianlin Liu.

**Writing – review & editing:** Zhen Meng.
